# Nonspecific synaptic plasticity improves the recognition of sparse patterns degraded by local noise

**DOI:** 10.1038/srep46550

**Published:** 2017-04-20

**Authors:** Karen Safaryan, Reinoud Maex, Neil Davey, Rod Adams, Volker Steuber

**Affiliations:** 1 Centre for Computer Science and Informatics Research, University of Hertfordshire, College Lane, AL10 9AB Hatfield, United Kingdom; 2 Department of Physics and Astronomy, Knudsen Hall, University of California, Los Angeles CA, 90095-0001, USA; 3 Department of Cognitive Sciences, Ecole Normale Supérieure, rue d’Ulm 25, 75005 Paris, France

## Abstract

Many forms of synaptic plasticity require the local production of volatile or rapidly diffusing substances such as nitric oxide. The nonspecific plasticity these neuromodulators may induce at neighboring non-active synapses is thought to be detrimental for the specificity of memory storage. We show here that memory retrieval may benefit from this non-specific plasticity when the applied sparse binary input patterns are degraded by local noise. Simulations of a biophysically realistic model of a cerebellar Purkinje cell in a pattern recognition task show that, in the absence of noise, leakage of plasticity to adjacent synapses degrades the recognition of sparse static patterns. However, above a local noise level of 20%, the model with nonspecific plasticity outperforms the standard, specific model. The gain in performance is greatest when the spatial distribution of noise in the input matches the range of diffusion-induced plasticity. Hence non-specific plasticity may offer a benefit in noisy environments or when the pressure to generalize is strong.

A central paradigm of neuroscience is that memories can be stored by adapting the strengths of synaptic connections^1^. After learning, re-application of a stored pattern re-produces an associated pattern of neuronal activity. The details of the implementation can differ, according to the learning rule used, the extent of dendritic processing, and the response metric taken as output^2^,^3^.

It has been suggested that the inputs may first undergo a transformation to a sparse pattern in a higher-dimensional space (for review see ref. 4). In a binary classification task, such a transformation could make the input patterns that are to be associated with each one of the binary outputs linearly separable by a hyperplane^5^. An expansion of input space into a higher-dimensional space is indeed observed in many neural systems, most prominently in the granular layer of the cerebellar cortex where granule cells outnumber their afferent mossy fibres by at least two orders of magnitude^6^,^7^. The granule cells presumably generate sparse patterns of activity^8^,^9^,^10^,^11^ that they convey to the principal neurons or Purkinje cells (PCs) via their ascending axons and parallel fibres (PFs).

The apparent lack of feedback has inspired theorists to model the PC as a perceptron^3^,^11^,^12^,^13^ that stores patterns through long-term depression (LTD) of active PF synapses during conjunctive climbing-fibre input^14^,^15^,^16^. The sparse activity of the granular layer enhances the storage capacity of the Purkinje cell (defined as the number of PF patterns that can be stored without intolerable error)^3^,^9^,^17^,^18^,^19^,^20^.

Nevertheless, these two views, of the granular layer as generating an expansive sparse code and of the Purkinje cell as a binary classifier, have recently been challenged on both experimental and theoretical grounds^21^. Firstly, the transformation of a (dense) input pattern into a sparse pattern is a process that is very sensitive to noise in the input layer^4^. This transformation therefore requires an intermediate (unsupervised) learning stage that maintains the clustering present in the input space^22^. Plasticity of the mossy-fibre-to-granule-cell connection may provide the neural substrate for this transformation^23^. Secondly, and more importantly, LTD at the parallel-fibre-to-Purkinje-cell synapse requires the production and release of NO by PFs^24^,^25^. This NO diffuses to neighboring synapses and compromises the synapse specificity of LTD^26^,^27^,^28^,^29^,^30^,^31^,^32^,^33^. A recent theoretical study predicted that such non-specific plasticity would be detrimental for memory^34^.

Hence both the lack of specificity at the input stage (pattern noise), and the lack of specificity of the learning rule (leakage of plasticity), are expected to affect memory storage and recall. In the present study we used computer simulations and mathematical analyses of Purkinje cell models with different degrees of complexity and biological realism to examine whether both drawbacks could compensate each other, that is, whether nonspecific LTD could make pattern recognition more robust in the presence of local spatial noise.

## Results

We examined the effect of leakage of plasticity (nonspecific LTD, or nsLTD) on the recognition of sparse, binary and stationary input patterns disrupted by local noise, in both a linear artificial neural network unit (further called ANN unit) and a morphologically realistic conductance-based Purkinje cell (PC) model (Table 1).

These models are described in detail in the Methods section. Briefly, the response *r* of the simple ANN unit was given by the inner product of the synaptic weight vector ***w*** and the input pattern vector ***x***:


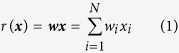


The multi-compartmental PC model^35^,^36^ contained a morphologically realistic representation of the dendrite and ten different types of voltage- and ligand-gated ion channels that were modeled using Hodgkin-Huxley-type equations. The model received continuous background input through excitatory PF and inhibitory interneuron synapses, and was active at a baseline rate of 48 spikes per s.

**Figure 1:**
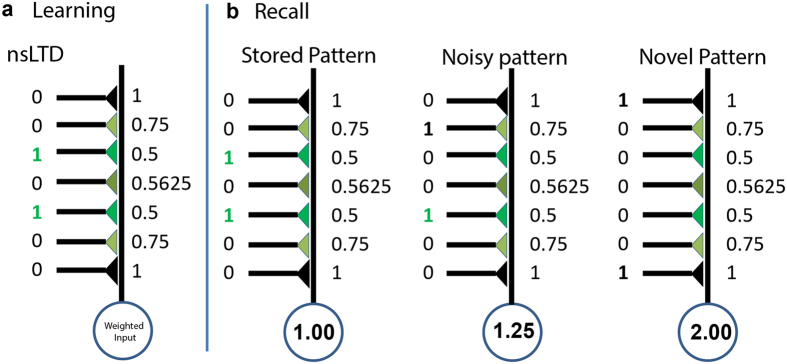
Pattern recognition in the presence of nonspecific LTD. The pattern recognition simulations involved two steps: learning (**a**) and recall (**b**), illustrated here for an ANN unit with, on its virtual dendrite, a nearest-neighbor-only leakage between the synapses. Each cartoon represents part of the virtual dendrite, with binary inputs arriving on the left via synapses that have color-coded weights with values indicated on the right. (**a**) Learning phase showing the array of weights after storing the single pattern on the left. All weights were initialized at unity, and were depressed following the leakage rule given in Eq. 7. Note the spread of LTD to the adjacent synapses. (**b**) Recall phase showing the readout of a stored, noisy stored, and novel pattern. The weights were fixed during recall, and the output at the soma was calculated as the inner product of weight vector and pattern vector. The noisy stored pattern was identical to the stored pattern except for one randomly selected on-bit that was shifted to a nearest-neighbor position.

The input patterns had *N* = 14,740 or 147,400 bits, one for each afferent PF, of which a randomly chosen subset (between 0.35 and 5.6%) was ON. A hundred such patterns were stored by LTD of the PF synapses using one-shot supervised Hebbian learning^5^,^17^ (Fig. 1a). (In the Mathematical Appendix in Supplementary Information, we show analytically that slightly potentiating the non-depressed synapses does not alter the characteristics of the learning rules).

In most simulations, the leakage of plasticity and the pattern noise were local, and could either be limited to a fixed radius of up to three nearest neighbors along the dendritic shaft (further called the 1D neighbor relationship) (Fig. 1b), or could show a volume spread according to a Gaussian distance profile (the 3D neighbor relationship). The same neighbor rules were used to select the noisy bits in noisy versions of the stored patterns. For the 3D relationship, the leakage of plasticity and pattern noise could spread with the same profile or show a mismatch.

**Figure 2:**
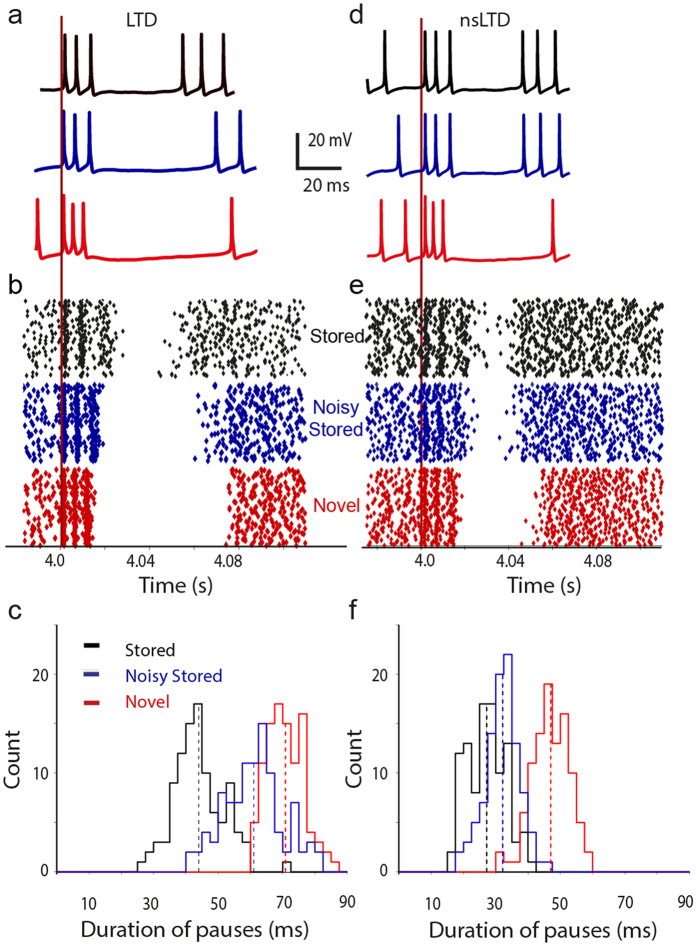
Pattern recognition in the biophysical PC model. The learning algorithm was either standard LTD (**a**–**c**, left column) or nonspecific LTD (**d**–**f**, right column). After learning, three sets of 100 patterns were presented. The first set contained patterns that had been stored (black), the second set contained noisy versions of the stored patterns (noise level of 50%, blue) and the third set consisted of completely novel random patterns (red). The pattern or PF stimulus was presented after 4 seconds of spontaneous activity (vertical red line). (**a**,**d**) Example membrane potential traces. (**b**,**e**) Raster plots of spike times for 100 different patterns of each category. (**c**,**f**) Response distributions as histograms of pause duration.

The pattern recognition performance was measured by comparing the responses to the 100 stored patterns (or 100 noisy stored patterns) to those to 100 novel random patterns. To this end, neuronal responses were quantified as the weighted input sum for ANN units (see Equation 1 and Fig. 1b) and as the duration of the pause in firing following the pattern-evoked burst for the PC model (see Fig. 2a,b and d,e and ref. 3). Figures 2c,f show examples of the response distributions of pauses evoked in the PC model.

Clearly, nsLTD (*right* column) enhanced the separation between the responses to noisy stored patterns (*blue*) and novel patterns (*red*), while decreasing the separation between stored and noisy stored patterns (*black* versus *blue*) and, to a lesser extent, the separation between stored and novel patterns (*black* versus *red)*. In the following sections, this phenomenon will be compared in a quantitative manner for ANN units and PCs, and for 1D and 3D synaptic neighborhood relationships.

The difference in response distribution (stored or noisy-stored versus novel), that is, the pattern recognition performance, was then quantified using a signal-to-noise ratio (s/n)^37^


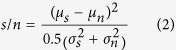


where *μ*_*s*_ and *μ*_*n*_ are the mean values and 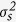 and 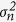 the variances of the responses to stored and novel patterns, respectively.

### Pattern recognition in an ANN unit with a 1D synaptic neighborhood function

**Figure 3:**
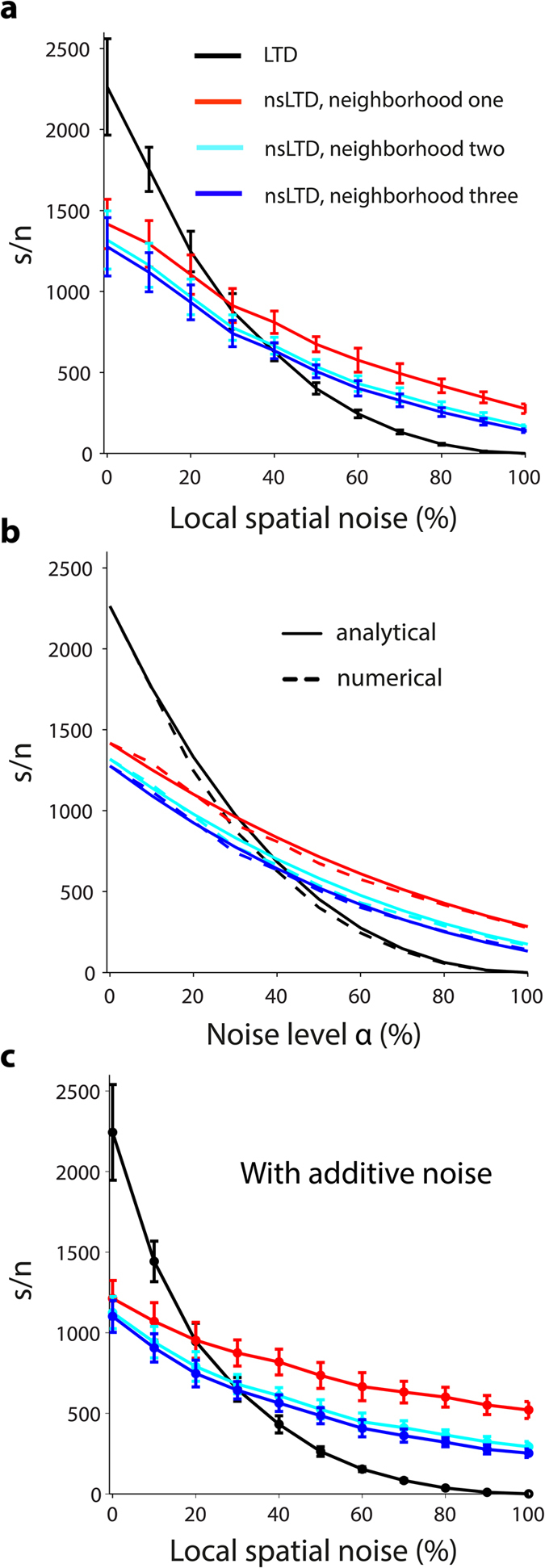
Pattern recognition in an ANN unit with a 1D synaptic neighborhood relationship. (**a**) The signal-to-noise ratio on the vertical axis was calculated from the response distributions to 100 novel (non-trained) patterns versus 100 stored patterns (zero noise data points located on vertical axis), or 100 novel patterns versus 100 noisy versions of the stored patterns. The degree of pattern noise on the horizontal axis expresses the percentage of ON-bits that had been moved to a neighbor, for different radii of the neighborhood function. The learning rule was either standard LTD (black curve) or nonspecific LTD with exponential spread to one (red), two (cyan) or three (blue) nearest neighbors on either side along the virtual dendrite. Note that here the same neighborhood relations applied for the spread of pattern noise and the leakage of LTD. Simulations of model ANN-1D in Table 1, *N* = 147,400 and pattern sparsity = 0.7%. (**b**) Comparison of the numerical simulation results from (**a**) (solid lines) with results from an analytical calculation of the signal-to-noise ratio for the same model and parameters (dashed lines) (see main text, and Mathematical Appendix in Supplementary Information file). (**c**) Simulation of the same ANN as in (**a**), but with combined standard and additive noise. The noise level on the horizontal axis now represents both the percentage of displaced ON bits in a pattern, as in (**a**), and the percentage of ON bits added. For instance, for patterns with 1000 ON bits, a noise level of 20% indicates that 200 ON bits had been swapped with OFF bits in their neighborhood, and that, in addition, 200 ON bits were newly added in the same neighborhood.

Figure 3a plots, for varying degrees of pattern noise, the signal-to-noise ratio of the simulated responses of a linear ANN unit to 100 novel versus 100 noisy stored patterns, each pattern consisting of *N* = 147,400 bits of which 1000 bits (0.7%) were ON.

In the absence of noise (0% on the horizontal axis), standard LTD was always more effective than nonspecific LTD, by a factor of almost 2. However, with increasing local noise levels, the performance fell more sharply for standard LTD, in such a way that above noise levels of 30–40%, nsLTD outperformed LTD. In these simulations, the leakage of LTD and the spread of noise had been matched, and decayed exponentially to a fixed number of one, two or three neighbors (ANN-1D, see Table 1 and Methods Equation 7).

### Analytical calculation of the signal-to-noise ratio

To better understand the results of the numerical simulations of the ANN unit we derived the signal-to-noise ratio analytically (see Mathematical Appendix in Supplementary Information). Figure 3b plots a comparison of the analytical and numerical results (numerical results as in Fig. 3a). The complete derivation given in the Appendix shows that, in the presence of nsLTD and for a neighborhood of 1, the relationship between the signal-to-noise ratio and the fraction *α* of noisy bits in a pattern can be approximated by (Appendix Eq. A10):


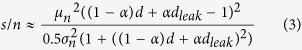


where *d* = *0.5* is the depression factor for activated synapses (ON bits in a pattern), and *d*_*leak*_ is the nsLTD depression factor in the neighborhood, set for example to 0.75 for nearest neighbor synapses in the 1D neighborhood function. For specific LTD, there was no additional depression in the neighborhood of activated synapses and *d*_*leak*_ was equal to 1. As a consequence, the curves describing the relationship between noise level *α* and signal-to-noise ratio had a shallower slope when the patterns had been stored with nsLTD (Fig. 3b).

In the absence of noise (for *α* = 0), the signal-to-noise ratio is given by


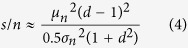


The value of the ratio 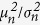 in Eqs 3 and 4 can be derived analytically by assuming that the number of times a synapse is hit by an ON bit in a pattern follows a Poisson distribution (see Appendix Eqs A11–A20). In our simulations and analyses nsLTD led to a smaller ratio 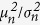 than specific LTD, which meant that nsLTD resulted in a smaller signal-to-noise ratio in the absence of noise (Appendix Eq. A23), and the s/n curves for LTD and nsLTD crossed each other at a particular noise level *α*.

### The learning rule is robust to additive noise

The present standard LTD learning rule, in which the depression of synapses is divisive and follows a geometrical progression (see Methods Equation 6), is a further elaboration of the learning rule used in the Willshaw associative nets^38^. A characteristic of these nets is that the patterns always have the same arity (density of ON bits), and it is well known that presenting patterns with fewer or more ON bits than the learned pattern will affect pattern recognition (see Supplementary Figure S1). There is one situation, however, where nsLTD offers an additional benefit: when the supernumerous synapses are activated within the neighborhood of the pattern’s ON bits (Fig. 3c). Such patterns with local additive noise correspond more closely to the clustered patterns of PF activation observed after peripheral stimulation^39^. In that case, the ANN trained with nsLTD weights the additional ON bits by depressed synapses, whereas specific LTD, which does not have a neighborhood function, cannot tell them apart from random synapses. Nonspecific LTD now starts outperforming specific LTD at 20% noise levels (Fig. 3c), as compared to 30% in the absence of additive noise (Fig. 3a).

### Pattern recognition in the PC model with a 1D synaptic neighborhood function

**Figure 4:**
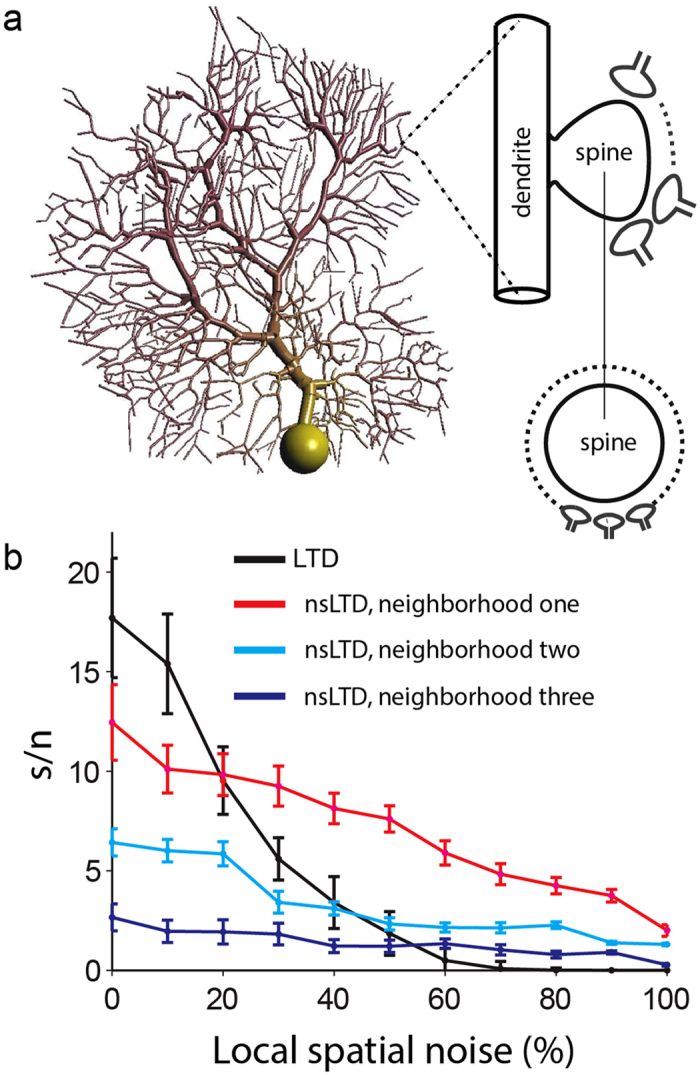
Pattern recognition in the PC model with a 1D synaptic neighborhood relationship. (**a**) Schematic representation of the PC-1D model, having one spine on each of its 1474 spiny dendritic compartments. In this simplified architecture, each spine received 100 individually weighted synapses from 100 PFs, hence the pattern size was *N* = 147,400. On each spine the synapses were arranged in a ring in order to define a neighborhood relation between them. (**b**) Pattern recognition performance, using the same format as used in Fig. 2. Simulations of model PC-1D in Table 1.

Very similar effects of local noise were observed for the biophysical PC model displayed in Fig. 4a.

When plasticity and noise spread only to the nearest neighbors (*red* curve), nsLTD already outperformed standard LTD at a noise level as low as 20% (Fig. 4b). Note that overall, the performance of the PC was an order of magnitude lower than that of the ANN unit (compare Figs 3 and 4), but this was partially a consequence of the different metrics used to characterize the responses (see Methods).

### Pattern recognition using 3D synaptic neighborhood functions

**Figure 5:**
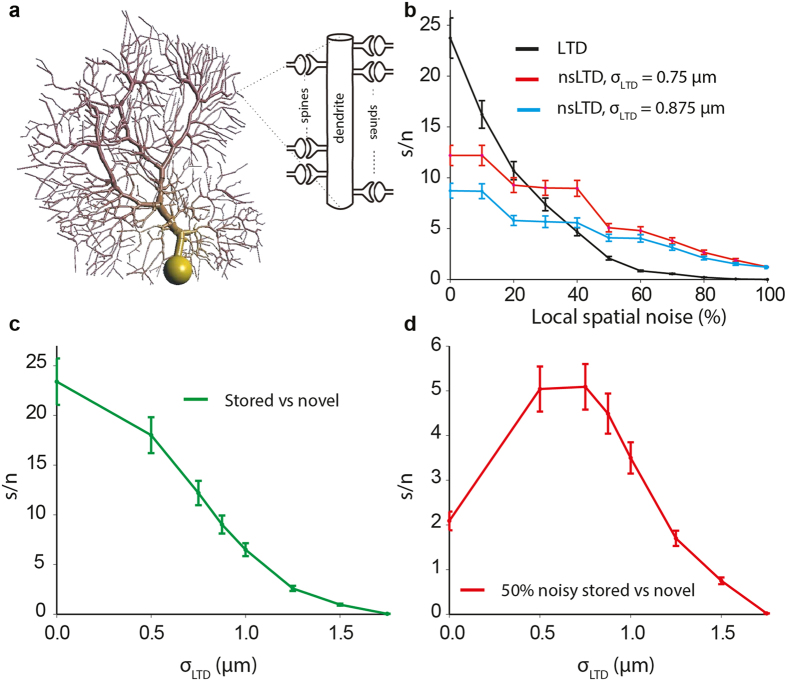
Pattern recognition in the PC model with a 3D synaptic neighborhood relationship. (**a**) Schematic representation of the PC-3D model, having ten spines on each of its 1474 spiny dendritic compartments. The ten spines were equally spaced along the compartmental axis, with their necks oriented in a random direction perpendicular to the axis. In this simplified architecture, each spine received one weighted synapse from one PF, hence the pattern size was *N* = 14,740. (**b**) Pattern recognition performance for LTD and nsLTD, using the same format as used in Figs 3 and 4b. Two spatial spreads of nsLTD are compared (expressed by the standard deviation σ_LTD_ of the Gaussian kernel, see Methods) and in each case σ_noise_ matched σ_LTD_. (**c**) Effect of varying the spatial spread of nsLTD (σ_LTD_) in the absence of pattern noise. (**d**) Effect of varying the spatial spread of nsLTD (σ_LTD_) for 50% noisy patterns with σ_noise_ fixed at 0.75 μm. Simulations of model PC-3D in Table 1.

In order to be able to implement more biologically realistic 3D Euclidean distances (as opposed to 1D nearest neighbor relationships) between PF synapses in the PC model, we reduced the number of PF inputs to *N* = 14,740, but made each PF innervate a unique individual spine by increasing the number of spines from 1 to 10 on each dendritic compartment (Fig. 5a). Note that this manipulation did not alter the input-output relationship of the model PC^36^ (Supplementary Fig. S2).

Moreover, Fig. 5b shows the same effects of local noise as observed above: a sharp decline in performance when noisy patterns were presented after training with standard LTD (black curve); a drop in performance with nsLTD in the absence of pattern noise; and an enhanced performance with nsLTD at local pattern noise levels above 20% (red and blue). In the absence of pattern noise, the performance declined monotonically with the leakage radius of nsLTD (Fig. 5c). In contrast, when noise was present, the performance was highest when the radius of leakage of LTD matched the spatial spread of the pattern noise (σ_LTD_ = σ_noise_ = 0.75 μm), falling off at smaller and higher radii (Fig. 5d).

### ANN units and the biophysical PC model compared

To further explore the quantitative difference between the effect of nsLTD in the ANN and the biophysical PC model, we introduced Euclidean distances between the synapses on the virtual dendrite of the ANN unit by using the same distances as those calculated between the corresponding synapses of the PC-3D model.

**Figure 6:**
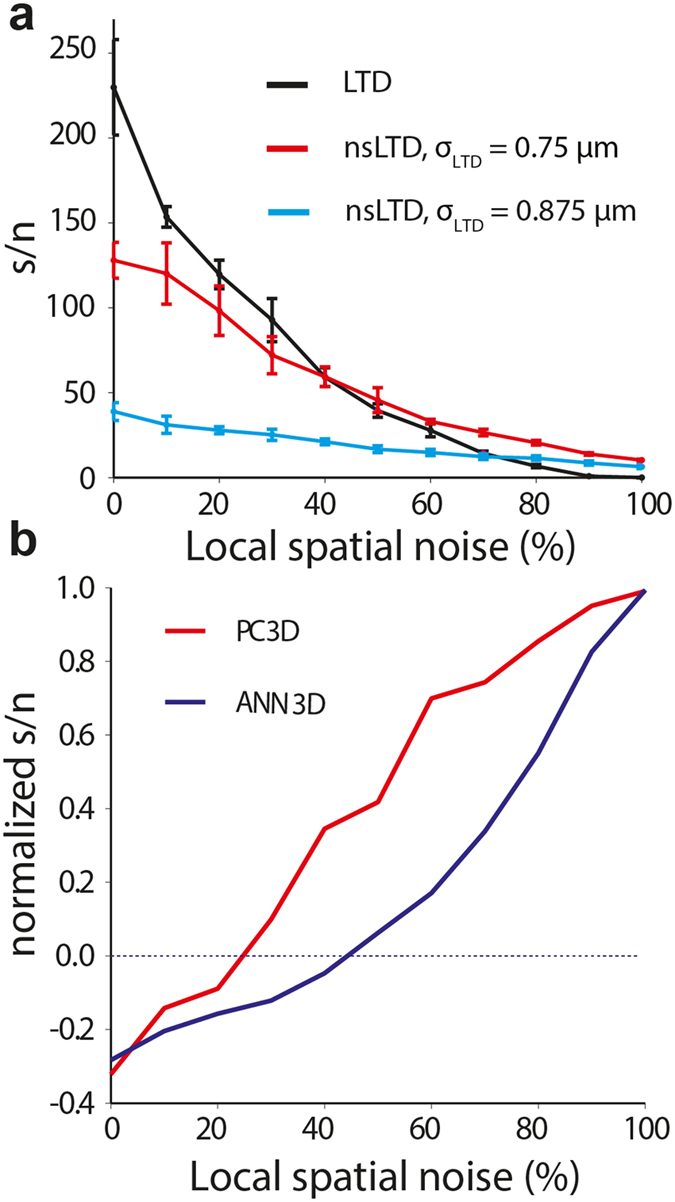
Pattern recognition performance of the PC-3D and ANN-3D models compared. (**a**) Pattern recognition performance of the ANN-3D unit. To introduce a 3D synaptic relationship in the ANN units, the same dendritic architecture as that of the PC-3D model was used. Data plotted using the same format as in Fig. 5b. (**b**) Relative performances for LTD and nsLTD, expressed as a normalized signal-to-noise ratio (see text), using σ_LTD_ = 0.75 μm. Positive (negative) values indicate that nsLTD performs better (worse) than LTD.

Figure 6 plots the performance of the ANN-3D unit in the same experiments as those plotted in Fig. 5b for the PC-3D model. Clearly, in the ANN-3D unit, for nonspecific LTD to outperform standard LTD, the patterns required higher noise levels than in the PC-3D model (about 40% versus 20%), and the gain in performance was lower. The difference between the PC model and the ANN unit is illustrated in Fig. 6b, which plots the gain in performance by nonspecific LTD relative to standard LTD, using the formula:


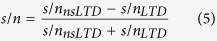


These results confirm that the larger robustness against noise introduced by nsLTD in the biophysical PC model compared to the ANN must be based on the non-linear synaptic integration in the PC model rather than the spatial distribution of inputs across the dendrite, which was the same for linear ANN-3D.

### Effects of pattern loading and sparsity

The observed beneficial effect of nonspecific LTD relative to standard LTD, illustrated in the previous sections after training with 100 binary patterns of 0.7% sparsity (0.7% ON-bits), could be extended to more dense patterns and to higher loadings in which both training and test sets contained greater numbers of patterns. For practical reasons, the effects of these two parameters were only examined in the ANN-3D model (ANN units to which the synaptic positions, and hence the inter-synaptic distances of the PC model had been copied, see above).

**Figure 7:**
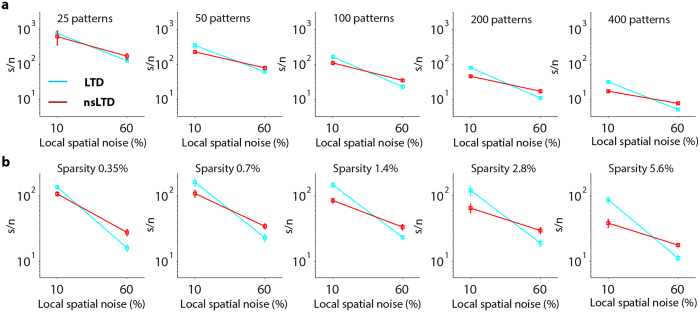
The effect of varying the number of stored patterns and their sparsity on pattern recognition performance of the ANN-3D unit. Learned patterns contaminated by either 10% or 60% of local noise were used to compare the effectiveness of LTD (*cyan*) and nsLTD (*red*). The unit had 14,740 inputs (see Table 1), and both nsLTD and pattern noise had a 3D spread of σ_LTD_ = σ_noise_ = 0.75 μm. (**a**) The loading was varied from 25 to 400 patterns, for patterns of 1% sparsity. (**b**) The sparsity, defined as the percentage (or density) of ON-bits in each pattern, was varied from 0.35 to 5.6%, for a fixed loading of *p* = 100 patterns.

Figure 7 compares LTD and nsLTD for two levels of local noise. At the lower noise level of 10%, standard LTD (*cyan*) was always better at telling apart noisy-stored patterns from novel patterns. In contrast, distinguishing very noisy patterns (60% noise level) from novel patterns was invariably better after training with the nsLTD rule (*red*). These conclusions held over the whole range of loadings (25 to 400 patterns, Fig. 7a) and pattern densities tested (0.35 to 5.6%, Fig. 7b). Supplementary Fig. S3 plots the s/n ratio as a function of the density of ON bits.

As compared to the ANN, the storage capacity of the biophysical PC model was rather limited (300–400 patterns in Steuber *et al*.)^3^. The ANN capacity has been calculated to amount to several thousands of patterns (see the work by Brunel *et al*.^9^, and our own calculations and Fig. A1 in Mathematical Appendix of Supplementary Information). On the other hand Fig. 6b showed that nsLTD was more effective in the model PC than in the ANN unit. It must thus be concluded that the PC has a limited capacity for the storage of uncorrelated patterns, but that this limitation is compensated by a greater ability to recognize noisy (hence correlated) patterns. It is also possible that the actual readout occurs downstream in cerebellar nucleus neurons, on which the outputs of about 40 PCs converge^40^.

### Effects of combined (ns)LTD and LTP

**Figure 8:**
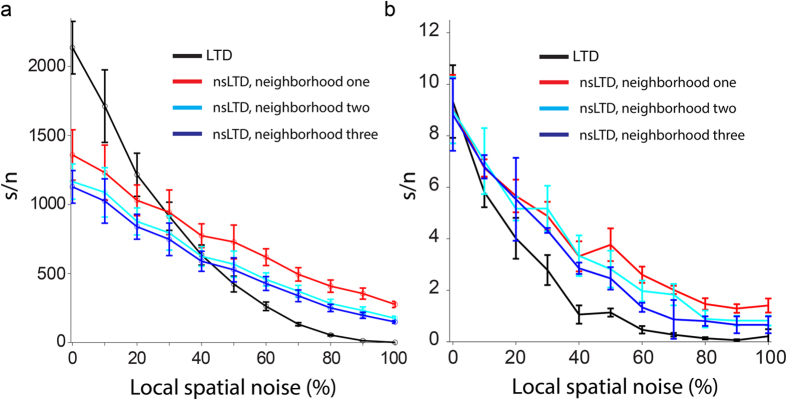
Adding LTP has little effect for the ANN unit but makes nsLTD superior to LTD at all noise levels in the PC model. Simulations of the ANN-1D (**a**) and the PC-1D model (**b**). Each time a pattern was stored, the non-active PF synapses were slightly potentiated so as to keep the overall weight constant. These LTP factors were calculated following Equation (A14b) of the Mathematical Appendix in Supplementary Information. They measured 1.0035 for specific LTD, 1.0072 for nsLTD with leakage to the nearest neighbors only, and 1.0091 and 1.0101 for nsLTD with leakage across two and three nearest neighbors, respectively.

In the previous simulations, the total change in synaptic weight was greater with nsLTD than with LTD because in addition to weights at active synapses, the weights in the neighborhood were also depressed (see Methods). To examine whether the difference in total weight change could affect our results, we compared the pattern recognition performance of the ANN-3D unit for LTD and nsLTD with equal mean synaptic weights after learning. As shown in Supplementary Fig. S4, this rescaling of the synaptic weights did not alter the signal-to-noise ratio, because the change in mean weight is compensated by an equivalent change of the variance. A candidate mechanism for weight homeostasis is LTP^28^,^41^ or slight potentiation of all inactive PF synapses each time a pattern is stored. The Mathematical Appendix predicts that adding LTP to the learning rule would not affect the performance of the ANN, under the assumption that the number of times a synapse is potentiated versus depressed follows a binomial distribution. This lack of an effect of LTP for pattern recognition by the linear ANN unit was borne out by numerical simulations (compare Fig. 8a to Fig. 3a).

In sharp contrast, the s/n ratio of the PC response was sensitive to the average synaptic weight, which determined not only the spontaneous spike rate but also the strength of the burst response and the duration of the subsequent pause. Interestingly, adding LTP to the learning rule in the default PC model made nsLTD equivalent or superior to LTD at all levels of pattern noise (Fig. 8b; see also Supplementary Fig. S5 for the weight distributions after training with combined LTP and nsLTD). The resulting weight homeostasis also prevented that the burst response would become too weak to be able to induce a pause (see raster plots in Supplementary Fig. S5), and, consequently, increased the number of patterns the PC could store with an s/n ratio >4 (from ~200 with simple LTD to more than 800 with combined LTP and nsLTD). This importance of LTP-induced weight homeostasis may explain the observed need for LTP in motor learning^42^,^43^,^44^.

## Discussion

Theories of learning in neural systems typically assume specific weight changes at activated synapses. In apparent contrast to this common assumption, it has been shown that in brain areas such as the cerebellum synaptic plasticity can spread to neighboring inactive synapses^26^,^27^,^28^,^29^,^30^,^31^,^32^,^33^. The presence of this kind of non-specific synaptic plasticity is expected to be detrimental for the recall of stored patterns^34^. We have investigated the storage and recall of input patterns in the presence and absence of non-specific long-term depression (nsLTD) in cerebellar PC models with different levels of complexity and biological realism. At noise levels above 20–30%, nsLTD outperformed standard LTD in a biophysical PC model in a standard pattern recognition task. Compared to the ANN units, which are optimal linear decoders, individual PCs performed rather poorly, but the recognition-enhancing effect of nsLTD manifested itself over a broader range of noise levels in the model PC than in the ANN unit (Fig. 6b, beyond 20% versus 40%). In addition, as has been shown before^45^,^46^ the signal-to-noise ratio will rise by several orders of magnitude when multiple PCs, trained by similar patterns, converge onto neurons in the cerebellar nuclei. Note that in the present model, the nuclear neurons would read out the patterns by an increase in their spike rate during the PC pause.

The leakage of LTD had to be restrained within a distance of about one μm for a positive effect of nsLTD to be observed (Fig. 5d). This spatial confinement is narrower than the spread of LTD over tens of micrometers originally reported in *in vitro* studies^26^,^29^,^32^. These *in vitro* studies may have overestimated the physiological action radius of NO, however, as a consequence of pharmacological (for instance bicuculline) or stimulation effects (bundles of PFs being fired). A more recent study, measuring NO-dependent LTP using a different stimulation protocol, observed a steep decline of heterosynaptic plasticity within 5–10 μm^47^,^48^. This is in closer agreement with modeling studies that simulated the NO concentration using the reaction-diffusion equation^33^. These studies found a strong nonlinear dependence of the action radius of NO on the diameter of the fiber by which it was released. For instance, for a fiber of 0.1 μm diameter, [NO] falls off to 50% at a distance of 2 μm^49^. Note that in mice, parallel fibers have an average diameter of 0.15 μm^50^. Moreover, in insects, spatial arrangements between NO and non-NO producing fibres have been shown to sharpen the resolution of NO effects^51^. Taken together, it must be concluded that the present nonspecific LTD learning rule operates much more locally than the (bidirectional) heterosynaptic plasticity rule that recently has been suggested to serve as a homeostatic control mechanism for the overall distribution of synaptic weights^52^.

The noise level of 20% at which nsLTD started to outperform standard LTD in the PC model (Figs 4b and 5b) may seem high, but taking into account the huge dimension of the input space (150,000 PF synapses on a rat PC), the probability that exactly the same pattern of parallel-fiber activity is generated twice during a lifetime seems to be vanishingly small^10^. Even though the synapses from mossy fibers onto granule cells are very reliable^8^,^53^, the mossy fibers to the same granule cells may convey not only peripheral inputs from different modalities^54^, but also information from neocortex that reaches the granular layer polysynaptically via the pontine nuclei, enhancing the probability of intervening noise.

An assumption of the present model is that noise preferentially spreads to neighboring parallel fibers, because the plasticity rule inevitably must be local and the spread of noise must match the leakage of LTD (Fig. 5d). There are no indications that neighboring PF synapses on a PC originate from neighboring granule cells in the granular layer^39^,^55^, the projections seem to be rather divergent. But in their recent technically ingenious study, Wilms and Häusser^39^ did find that behaviorally relevant stimuli excite clusters of neighboring parallel fibers, in spite of their being coded in a distributed fashion in the granular layer. It is therefore conceivable that local noise in stimulus space is propagated within clusters of parallel fibers, hence that the natural neighborhood relationships are preserved. At first sight, the clusters of co-activated PFs observed by Wilms and Häusser^39^ may be too large (median distance of 11 μm) for the very local action of nsLTD in the present simulations (Fig. 5), but the labeling of PFs was too sparse in this imaging study to reliably measure cluster size.

It should be noted that the findings of our present study do not depend on the specificity of the modulator involved. For instance, intracellular free Ca^2+^, and Ca^2+^-dependent synaptic signals, may invade neighboring spines along the dendritic shaft within a distance of 10 μm, not only in cerebellar Purkinje cells^29^,^56^, but also in hippocampal pyramidal cells^57^,^58^,^59^.

In summary, the present paper suggests that nonspecific synaptic depression evoked by nitric oxide diffusion to neighboring synapses may have a functional role. nsLTD made the response of a model Purkinje cell robust against noise in the precise location of the activated synapses. If this spatial noise or variability in synaptic activity reflects natural errors or variability in sensory signals or motor commands, nonspecific plasticity may be a mechanism for error correction and/or pattern generalization and completion. In the PC with 147,400 parallel fiber synapses, however, nsLTD provided a significant advantage only when the noise and leakage of plasticity were very local (on the order of magnitude of a micrometer). This spatial confinement may be below the experimental detection limit, and it may therefore be useful to extend this study to model neurons with smaller densities of synapses.

Leakage of plasticity is also at the heart of the formation of neuronal maps^60^,^61^ and of bio-inspired clustering algorithms like gas nets^62^ and volume transmission through diffusion of NO at parallel-fiber synapses^33^ or climbing-fiber synapses^63^ has been suggested to improve motor learning in robots. As a final remark, it must be admitted that a paradigmal cerebellar task such as eyeblink conditiong has recently been attributed to adaptive timing by intrinsic Purkinje cell mechanisms^64^,^65^.

## Methods

### Pattern recognition task

Single neurons were trained with a set of sparse static input patterns, and were then tested for their capacity to distinguish, by the strength of their response, learned from random novel patterns. The input patterns were uncorrelated and binary, one bit for each afferent, which was set to ON if the corresponding synapse was activated by the pattern. More particularly, we examined whether the leak of plasticity to neighboring synapses during the training phase generated robustness to local noise applied to the pattern during the test (recall) phase (Fig. 1).

### Neuron models

We simulated two categories of neuronal models (Table 1): artificial neural network (ANN) units and various versions of a biophysical model of a cerebellar Purkinje cell (PC).

The ANN units were simple linear summation units that generated as their output *r* the inner product of the synaptic weight vector ***w***and the pattern vector ***x*** (Equation 1, and Fig. 1a,b). As the patterns were binary, and the weights positive, the output of the ANN was a positive value. The number of synapses, and hence the pattern size *N*, was either 147,400 or 14,740 (see Table 1).

The biophysical Purkinje cell model^35^,^36^ consisted of a soma, and a dendrite of 1599 compartments, out of which 1474 were budded with spines that received AMPA receptor synapses from PFs. Since the number of spines on a single PC amounts to approximately 150,000 in rats^66^, and since each spine requires for its implementation a neck and head compartment, it was not practical to model all spines in the present learning paradigm. Instead, two variants of the PC model were simulated (Table 1). PC-1D received the full set of 147,400 PF afferents, but these were lumped into groups of 100, each group innervating the same single spine that a compartment was equipped with (Fig. 4a). PC-3D had a more realistic configuration of spines, each spine being innervated by a unique PF, but their number (and hence pattern size) was reduced to *N* = 14,740 (Fig. 5a).

The PC model had an intrinsic spike rate, with all synapses blocked, of 70 s^−1^. For the present *in vivo* simulations, each of its GABA_A_ receptor synapses was randomly activated at 1 Hz, and the background PF spike rate was set at 0.28 Hz (2.8 Hz in the PC-3D model) so as to confer to the PC a spontaneous activity of 48 spikes s^−1^.

### Neighborhood functions

Both the leakage of plasticity and the spatial spread of pattern noise required defining a neighborhood function. This could be one-dimensional (1D) or three-dimensional (3D). In the 1D case (ANN-1D and PC-1D, see Table 1), each synapse onto an ANN unit, as well as each of the 100 PFs converging onto the same PC spine, was given a fixed index in a ring array, by which also its nearest and next-nearest neighbors were defined (see Fig. 4a). In the 3D case, in contrast, the actual architecture of the PC dendrite was used to calculate Euclidean distances between spines, or, equivalently, between the bits in a pattern (PC-3D, see Fig. 5a). In ANN-3D, the synapses were mapped onto the PC morphology, but the output was calculated, as for ANN-1D, as a weighted sum.

Once the 3D distances between synapses were determined, leakage of plasticity was modelled as a 3D Gaussian kernel of distance, and the same kernel (albeit not necessarily with the same width) was used to represent the decaying probability with distance of a pattern bit being switched ON erroneously by noise (see below).

### Synaptic plasticity rules

In actual PCs, PF synapses undergo LTD when their activation is temporally associated with a dendritic complex spike, evoked by the activation of the PC’s climbing fibre. This climbing fibre signal functions as a teacher, but was not explicitly implemented in the present study.

In simulations with specific or standard LTD (briefly ‘LTD’), only those PFs actually activated were depressed. We here used a depression factor of *d* = 0.5 (the effect of *d* becomes explicit in the Mathematical Appendix). Hence the weight *w*_*i*_ of synapse *i*, after storing *n* patterns (indexed by *j*), was equal to


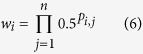


where *p*_*i,j*_ = 1 if the *j*th pattern had an ON bit at synapse *i*, and zero otherwise. It has here implicitly been assumed that the weights started from a value of 1. For the PC model, *w*_i_ was the factor with which the initial peak synaptic conductance of 200 pS had to be multiplied to obtain the resulting conductance of the depressed PF-to-PC synapse.

In simulations with nonspecific LTD (nsLTD), the depression spread to neighboring PF synapses even if these were not active during climbing fibre activation. For the 1D-neighborhood function, weights were updated as follows


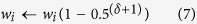


where *δ* is the distance of synapse *i* to the active PF synapse, counted as path length on the ring array, hence *δ* = 1 for the two nearest-neighbor synapses, *δ* = 2 for the two second-nearest neighbors, etc. Usually the depression was limited to up to three nearest neighbors on either side (see Fig. 4b).

When the 3D neighborhood function was used, all the synapses of the model were adapted by a factor equal to 0.5 times the value of a Gaussian distance kernel centred at the active PF synapse


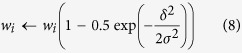


where *δ* is the distance to the active synapse in 3D space, and *σ* is the standard deviation of the Gaussian.

### Patterns and noise

As stated above, the patterns were uncorrelated and sparse; a sparsity of 0.007 was used for most of the simulations (the exception being control simulations with varying sparsity, see Fig. 7), meaning that 0.7% (1000 out of 147,400 or 100 out of 14,740) of the pattern bits were ON, and hence 0.7% of the synapses activated by each pattern. Noise was applied as a percentage of ON-bits being displaced from their original position as given in the trained pattern. After the bit to be displaced (or synapse) had been randomly selected, it was assigned to a neighbor according to the defined neighborhood relationship, 1D or 3D. In most simulations, the probability of a neighbor being selected as target for the displaced bit was proportional to the degree of nsLTD applied to the corresponding synapse. Figure 5d examines the effect of disparity between the local spread of noise and LTD.

Hence, in the case of one-dimensional nsLTD (in ANN-1D or PC-1D) with leakage to only the nearest neighbor on either side, the probability for each neighbor of being selected to activate its input (switching from OFF to ON) would be equal to 0.5. For a two-nearest-neighbor leakage, these values would be 0.33 (for each nearest neighbor) and 0.17 (for each next-nearest neighbor), etc.

To select neighbors for pattern noise in three-dimensional nsLTD (in ANN-3D or PC-3D), the cumulative distribution function was calculated of the Gaussian neighborhood function centred at the synapse selected for noise (the synapse being switched from 1 to 0). After this, a number was drawn randomly from a uniform distribution over the [0, 1] real interval and inversely mapped, by the cumulative distribution function, onto the domain of synapses. This way, the probability of a pattern bit (synapse) being switched from 0 to 1 by the noise was proportional to its Gaussian distance from the selected synapse (the central bit was prohibited from being selected).

### Output metrics

The pattern recognition performance of a neuron model was assessed by the signal-to-noise ratio of its responses to 100 stored versus 100 novel patterns. The selected response criterion was different for ANN units and the PC model. For an ANN unit, the response was its level of excitation, calculated as the weighted sum of inputs it received (the inner product of weight and pattern vector, Equation 1). For the biophysical PC model, which generated action potentials, the most sensitive response metric^3^ was the duration of the pause in firing following the initial burst response to the pattern and before spontaneous spiking resumed (see Fig. 2a,d).

From the distribution of the obtained responses (Fig. 2c,f), a signal-to-noise ratio was calculated as in ref. 67:


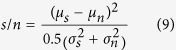


which is the square of the difference in mean response between 100 stored and 100 novel patters, divided by the mean of their variances. For robustness, the whole procedure of learning and recognition was repeated 10 times to obtain an average *s/n* and a standard deviation indicated by error bars in the figures.

### Implementation

The ANN model and all analyses were implemented in Matlab (The Mathworks). The PC model was converted from its original Genesis code to Neuron^68^. The simulations were run on the University of Hertfordshire Science and Technology Research Institute high-performance computing facility.

## Additional Information

**How to cite this article**: Safaryan, K. *et al*. Nonspecific synaptic plasticity improves the recognition of sparse patterns degraded by local noise. *Sci. Rep.* **7**, 46550; doi: 10.1038/srep46550 (2017).

**Publisher's note:** Springer Nature remains neutral with regard to jurisdictional claims in published maps and institutional affiliations.

## Supplementary Material

Supplementary Information

## Figures and Tables

**Table 1:** The four models used in the present study.

	Neuron model	Neighborhood relationship	Number of inputs	Number of spines	Figures
ANN-1D	linear unit	1D	147,400	—	1, 3, 8, S1
ANN-3D	linear unit	3D	14,740	—	6, 7, S3, S4
PC-1D	biophysical PC model	1D	147,400	1,474	2, 4, 8, S2, S5
PC-3D	biophysical PC model	3D	14,740	14,740	5, 6, S2

Abbreviations: PC, Purkinje cell; ANN, artificial neural network unit; 1D (3D), one(three)-dimensional.
