# Supplementary material for: Nonspecific synaptic plasticity improves the recognition of sparse patterns degraded by local noise
Source: Sci Rep. 2017 Apr 20;7:46550. doi: 10.1038/srep46550 (PMC5397845; doi:10.1038/srep46550)
Supplement: Supplementary Information [file srep46550-s1.pdf]

Nonspecific synaptic plasticity improves the recognition of sparse patterns degraded by local noise

Karen Safaryan,[1,2] Reinoud Maex,[1,3*] Neil Davey,[1] Rod Adams,[1] and Volker Steuber[1]

Contents of Supplementary Information

Figure S1	page 2
Figure S2	page 3
Figure S3	page 4
Figure S4	page 5
Figure S5	page 6
Supplementary Discussion	pages 7-11
Mathematical Appendix with Figure A1	pages 12-25
Figure A1	page 25
Supplementary References	page 26

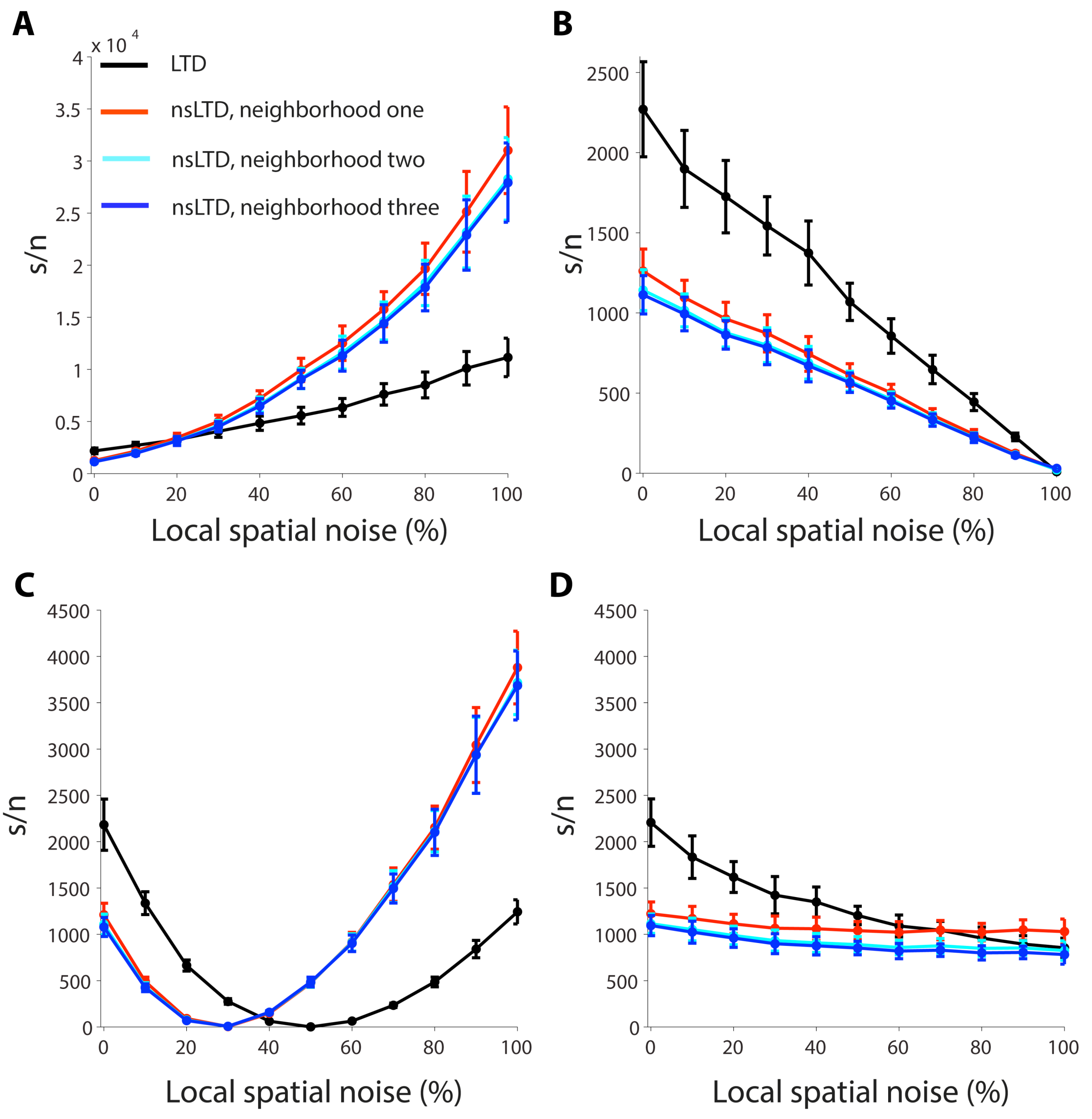

Figure S1 | Both specific (black traces) and nonspecific (colored) LTD lose their effectiveness in the presence of purely subtractive or additive pattern noise. In subtractive noise (A and B) the recall pattern had fewer ON bits than the stored pattern by the percentage indicated on the horizontal axis. In additive noise (C and D), the same percentage of ON bits was added in the neighborhood of genuine ON bits. Panels A and C represent the case that the number of ON bits changed only in the recall patterns, not in the novel patterns they were compared with (where it remained equal to that of the stored patterns). In B and D the arity of the novel patterns was changed in parallel with that of the recall patterns. Nevertheless, also here the advantage of nsLTD was lost.

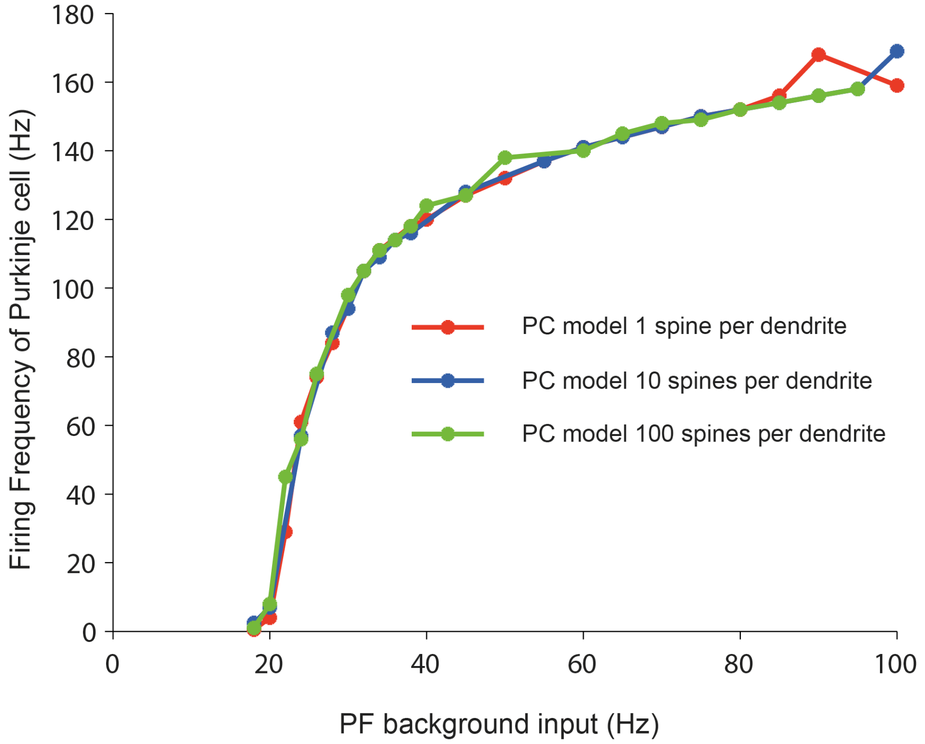

Figure S2 | Frequency-response curves of the Purkinje cell model with three different configurations of dendritic spines. Simple-spike frequencies are plotted for varying frequencies of background asynchronous PF excitation balanced with 1 Hz inhibition. In the complete absence of synaptic input, the model PC spiked spontaneously at 70 spikes s^{-1}.

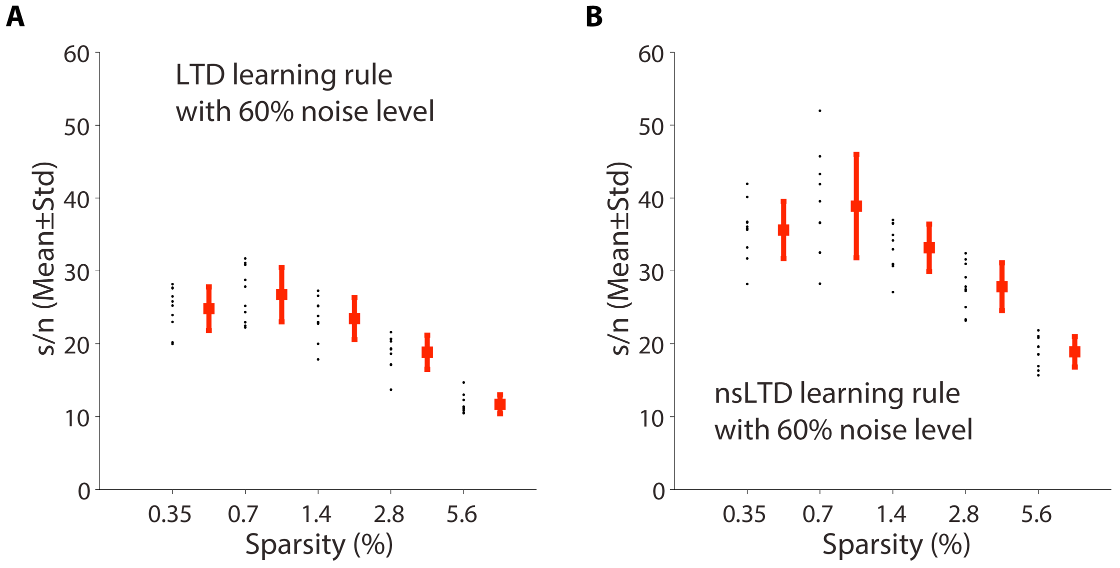

Figure S3 | The effect of pattern sparsity (the frequency of ON bits) on the signal-to-noise ratio in the ANN-3D model (elaboration of data shown in Fig. 7B). Blue dots are responses to single trials, each comparing responses of 100 noisy stored to 100 novel patterns, after training with specific LTD (A) or nonspecific LTD (B). Red dots and error bars (offset for clarity) are mean and standard deviation across the ten trials. There is an optimum at a density of ON bits of about 1 %. Note that this optimum is lost in the mathematical analysis (Fig. A1 in Mathematical Appendix), which is only approximate at very low loadings.

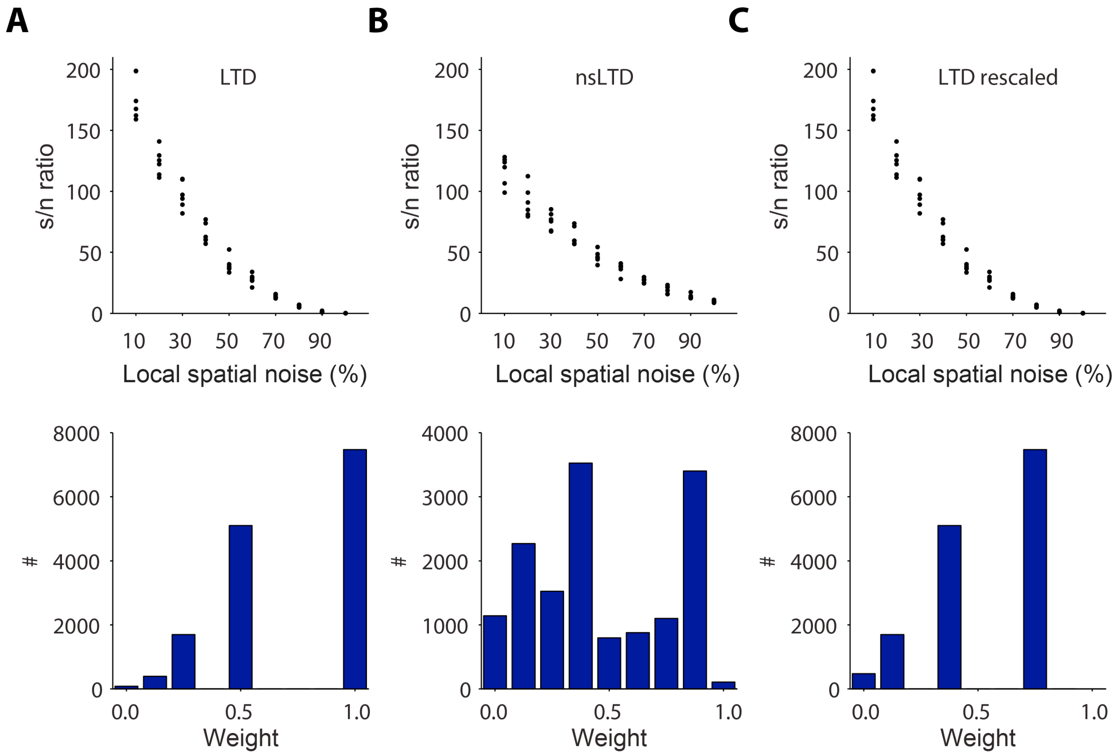

Figure S4 | Invariance of signal-to-noise ratio in the presence of scaled synaptic weights. (A, B) pattern recognition performance and synaptic weight distribution of the ANN-3D unit (see Table 1) in the presence of standard LTD (A) and nsLTD with σ_{LTD} = 0.75 μm (B). (C) pattern recognition performance and weight distribution for LTD with scaled synaptic weights so that the mean weight is the same as for nsLTD in (B). The mean weights on the histograms were 0.71 ± 0.31 (A), 0.54 ± 0.3 (B), and 0.54 ± 0.23 (C).

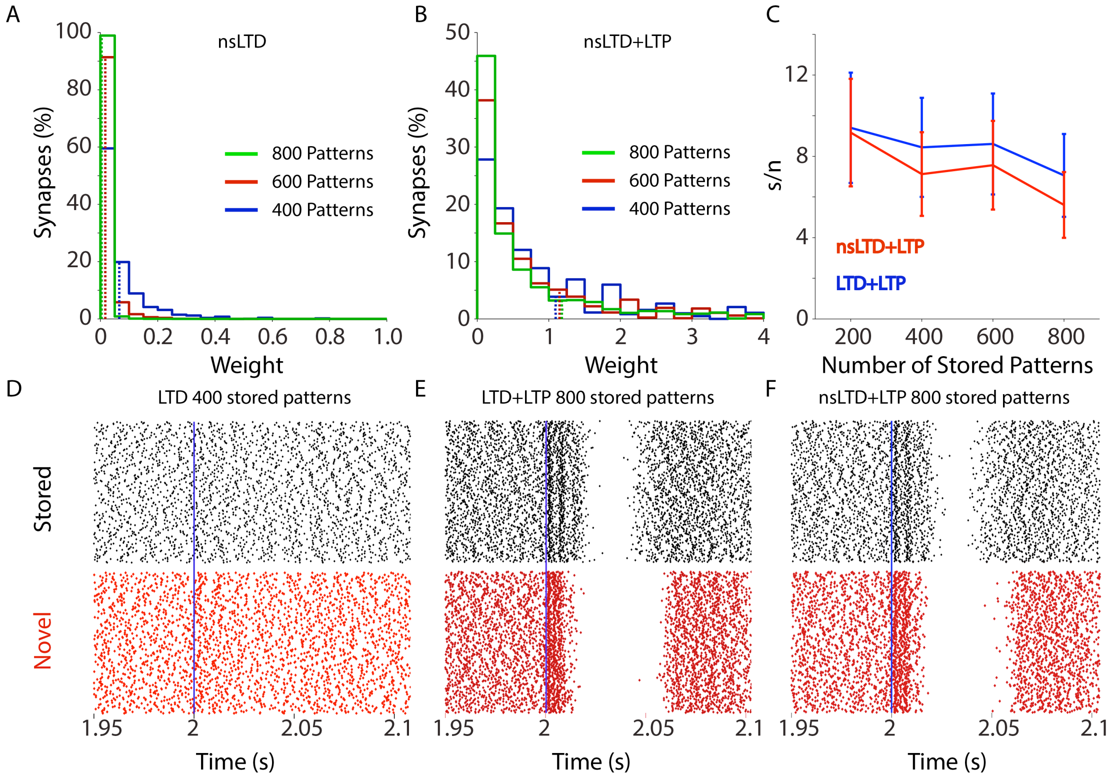

Figure S5 | Combining LTP and nsLTD prevents blunting of the burst-and-pause response of the biophysical PC model and enhances its storage capacity. (A, B) Weight distributions after storage of p = 400, 600 or 800 patterns with nsLTD alone (A) or nsLTD + LTP (B). In the nsLTD rule plasticity leaked only to the nearest neighboring synapses, and the LTP factor was set at 1.0072 so as to keep the overall weight approximately constant (see Eq. (A14b)). (C) Whereas LTD was superior to nsLTD for storing non-noisy patterns (see Figs. 4B and 5B in main text), both become equivalent when LTP of non-active PF synapses is added to the learning rule. (D-F) Raster plots of the PC responses to 100 stored (*black*) and 100 novel patterns (*red*) for three different conditions: p = 400, only LTD (D); p = 800, LTD + LTP (E); p = 800, nsLTD + LTP (F).

Supplementary Discussion

Here we discuss the robustness of the nonspecific learning rule (nsLTD) and its relevance for Purkinje cell (PC) physiology. A complete parameter analysis is impracticable with the full biophysical PC model. For this reason we also refer to analytical results of the Mathematical Appendix, to simulation data of the ANN, and to previous work.

Nonspecific versus specific LTD

In the absence of pattern noise, nsLTD performed worse than LTD (see the drops in s/n ratios up to 50 % at zero noise levels in Figures 3-7). The analytical expression for this can be found in Eq. (A23) in the Mathematical Appendix. ANNs trained with LTD will consequently be able to store more patterns than after training with nsLTD. For sufficiently noisy patterns, however, this relationship inverses, and the storage capacity is greater with nsLTD. Because the noise was always local, nsLTD can be said to increase the storage capacity for local noisy (hence correlated) patterns, whereas that of LTD will be higher for random, uncorrelated patterns. The performance of LTD versus nsLTD will consequently depend on the spatial structure of the noise.

Nonspecific LTD combined with LTP

Long-term potentiation (LTP) of PF synapses has been shown to be indispensable for the acquisition of motor behavior that relies on Purkinje cell activity (Schonewille et al., 2010). In section A4 of the Mathematical Appendix, the s/n ratio of the ANN was calculated analytically for the case that each non-depressed synapse is slightly potentiated so as to keep the total synaptic weight at its initial value. The dependency

of the s/n ratio on the number p of stored patterns was very similar to that obtained with LTD (or nsLTD) alone (compare Eqs. A20 and A20a, in both equations the s/n ratio has an exponential dependency on p in the denominator). Hence adding LTP will approximately amount to multiplying all the synaptic weights by a constant factor, and this operation does not affect the s/n ratios for the ANN (Fig. S4) as the constant factor appears in both the numerator and the denominator of the formula of the s/n ratio. PCs however have a spike threshold, and for them not only the shape of the weight distribution is important (the relative weights), but also the absolute values. Previous simulations of the biophysical PC model showed a critical effect of the overall level of excitation (Fig. 4 in de Sousa et al., 2014). Figures 8 and S5 showed that weight homeostasis through LTP kept the pattern-evoked responses suprathreshold, and as such LTP may be indispensable for learning. The present model cannot explain, however, the residual motor learning that has been observed in the absence of LTD (Gutierrez-Castellanos et al., 2017), and which may rely partly on compensatory plasticity at interneuron synapses.

Sparsity of the patterns

The density or fraction of ON bits is an important parameter in several respects. Its value was varied in Fig. 7B for the ANN-3D model, and is denoted by the symbol f in the Mathematical Appendix. Figure A1 shows that the storage capacity decreases with f, especially when LTD progresses rapidly, meaning that each time a synapse is depressed, its weight is considerably reduced (symbol d in the legends indicates the factor by which the weight is multiplied each time). The storage capacity rapidly declines for $f > 0.1$, even with $d = 0.99$. Hence less than 10 % of the granule cells should be activated by a pattern. Even though recent imaging studies show non-sparse

activation of the granular layer, peripheral stimuli triggered Ca^{2+} transients in only 2.8 % of the parallel fibers in Wilms and Häusser (2015).

Another issue concerns the variability of the density of ON bits. When ON bits are added, or deleted, to the recall patterns, without a like change in the novel patterns against which they are tested, the s/n ratio declines rapidly (Fig. S1C). As is the case with most models of pattern recognition by Purkinje cells, our model too assumes that control mechanisms stabilize the average granule cell activity. In Marr's seminal model, this function was attributed to Golgi cell feedback (Marr, 1969), which may generate tonic inhibition of granule cells.. When, in contrast, stored patterns are always checked against novel patterns of the same arity (Supplementary Fig. S1 B and D), the decline in performance is less pronounced, but the advantage of nonspecific LTD is still lost. In the actual cerebellum, checking stored patterns against novel patterns of the same arity may be implemented, for instance, by comparing the response of the trained PC directly to the responses of naive PCs that were not trained to recognize the pattern in question. This comparison may be accomplished downstream in the deep nuclei, or within the cerebellar cortex via the Purkinje axons collaterals.

An especially advantageous case consists of the combination of regular noise with additive noise (Fig. 3C). In this case a certain percentage of ON bits is not only locally displaced, but an equal percentage of new ON bits is added in the neighborhood. Such spurious ON bits would correspond to the activation of small clusters, instead of singletons, of parallel fibers (Wilms and Häusser, 2015).

Silent synapses

In actual Purkinje cells and in the perceptron model, most of the synapses were silent at full storage capacity (Brunel et al., 2004). With the present LTD learning rule (with a geometric or divisive weight reduction), the storage capacity of the ANN was of the same order of magnitude as that of the perceptron model (Fig. A1 in Mathematical Appendix). In a previous study (Steuber et al., 2007), pattern recognition rapidly declined in the PC model when more than 200 patterns were stored with LTD alone. The present study (Fig. S5) shows that adding LTP extends the storage capacity to >800 patterns (panel C) for realistic weight distributions (panel B).

It is however impracticable at the moment to measure the maximal capacity of the biophysical PC model. As mentioned above, LTD should be taken very weak ($d > 0.9$) except when the patterns are very sparse (see Fig. A1). With such a large value of d, the s/n ratios were very low in the PC model, and too many trials would be needed to reach significance. It is not implausible further that the patterns are read-out by neurons in the deep nuclei, which receive convergent input from on average 40 PCs (Person and Raman, 2011). Simulations by us (Luthman et al., 2009) and others (Walter and Khodakhah, 2009) have borne out that the s/n ratio can be orders of magnitude greater at the level of the deep cerebellar nuclei than in their afferent Purkinje cells.

Bursting patterns and synchrony

When the pattern is conveyed by single PF spikes, temporal jitter can deteriorate pattern recognition performance, but only when the jitter window is greater than 7 ms (see Figure 9D in Steuber et al. (2007)). In awake animals granule cells fire in bursts, and this enhances synaptic transmission to the PC (van Beugen et al., 2013). The

observed loss of temporal precision will have little effect on pattern recognition performance, however, when the pause following the burst is taken as metric. In a previous study by our group, pattern recognition by an active multi-compartmental neuron had been optimized using genetic algorithms (with bursting as a free parameter), and the optimized models always preferred brief bursts as input (de Sousa et al., 2015).

Synaptic drift and noise

Panel C of supplementary Fig. S4 considered the case of multiplying (up-scaling) all synaptic weights by the same factor. Comparison with panel A shows that this does not change the s/n ratio, the reason being that the factor appears in both the numerator and the denominator of the s/n formula. So the model is robust against global weight drifts. The model is also robust against variations in the strength of AMPA receptor mediated synaptic currents (quantal variance) and variations in the depth of LTD (factor d in Mathematical appendix, which has a value of 0.5 in all simulations in the main text), as shown previously (Fig. 9 C in Steuber et al. (2007)).

Intrinsic Purkinje cell activity and feed-forward inhibition

When the pause duration is taken as metric, pattern recognition improves when the intrinsic PC spike rate is high, or the inhibition / excitation ratio low (see Figures 7 and 9 A, B in (Steuber et al., 2007)). Hence intrinsic PC plasticity, and the level of inhibition, may further modulate the pattern recognition performance.

Mathematical Appendix: analytical calculation of the signal-to-noise ratio

We here give an analytical calculation of the signal-to-noise ratio (*s/n*) of pattern recognition by the ANN unit. Recall that the signal-to-noise ratio compares the response distributions to (noisy) stored and novel patterns and is expressed as:

$$s/n = \frac{(\mu_s - \mu_n)^2}{0.5(\sigma_s^2 + \sigma_n^2)} \qquad \text{(A1)}$$

(for symbols, please see below) and that the ANN response *r* to a single pattern *x* is calculated as the inner product of the vector of (binary) pattern bits and the weight vector *w*

$$r(\boldsymbol{x}) = \boldsymbol{wx}\sum_{i=1}^{N} w_i x_i$$

We proceed in three steps:

A1. Semi-analytic calculation of the ANN response to noisy stored patterns,

A2. Calculation of the ANN response to novel patterns using specific LTD,

A3. Calculation of the ANN response to novel patterns using nonspecific LTD (nsLTD).

A4. ANN response after combined nsLTD and LTP.

The last section (A5) compares the s/n metric with the reliability metric used by Brunel et al. (2004).

Symbols d denotes the LTD depression factor (0.5 in Eq (6) of main text)

d_{leak} denotes the nsLTD depression factor in the neighborhood (0.75 for nearest neighboring synapses, 0.875 and 0.9375 for next- and next-next nearest neighbors in Eq (7) of main text)

N denotes the number of inputs

f denotes the fraction of ON bits in a pattern (density)

p denotes the number of patterns to be stored

μ_s (μ_n) denotes mean response to stored (novel) patterns, and σ_s (σ_n) denotes their standard deviation

μ_w denotes mean of the weights distribution, and σ_w denotes standard deviation

α denotes the fraction of noisy bits in a pattern

A1. Semi-analytic calculation of the ANN response to noisy stored patterns

We here give semi-analytical expressions for the effect of noise on pattern recognition by an ANN trained with specific LTD (subsections A1.a and A1.b) or nonspecific LTD (A1.c).

We assume that the mean (μ_n) and standard deviation (σ_n) of the responses to novel patterns are known, and use these values to calculate the mean (μ_s) and standard deviation (σ_s) of the responses to (noisy) stored patterns. The calculation of μ_n and σ_n is postponed to sections A2 and A3.

A1.a Specific LTD: response to stored patterns

Let μ_n^{p-1} denote the mean response to a *novel* (non-stored) binary pattern after all but one of the total of p patterns have been stored. When the last pattern is being stored,

the active synapses (corresponding to the ON bits in this pattern) will be depressed by a factor *d.* Because the ANN response to a binary pattern is exclusively determined by the ON bits, the response to this last pattern must also be less by the same factor *d* than the response to a random novel pattern before this pattern had been stored, thus $\mu_s^p = d\mu_n^{p-1}$. The same reasoning can be made for every stored pattern (their sequence of storage is irrelevant, see Eq (6) of main text), so this relationship must hold in general.

Storing the *p*th pattern will change in turn the mean response μ_n^p to novel patterns, but because the patterns are sparse (0.7 % of ON bits) we can assume this effect to be small so that approximately $\mu_n^p \approx \mu_n^{p-1}$. Hence, suppressing the superscripts,

$$\mu_s \approx d\mu_n, \tag{A2}$$

irrespectively of the details of the weight distribution and the drawing strategy.

Similarly we can write for the standard deviation

$$\sigma_s \approx d\sigma_n, \tag{A3}$$

which holds again irrespectively of the details of the weight distribution and the drawing strategy, the latter having been taken already into account in the variable σ_n.

Substituting Eqs. (A2) and (A3) in (A1) yields for the s/n ratio of the response to stored patterns

$$\begin{aligned} s/n &= \frac{(\mu_s - \mu_n)^2}{0.5(\sigma_s^2 + \sigma_n^2)} \\ &\approx \frac{(d\mu_n - \mu_n)^2}{0.5(d^2\sigma_n^2 + \sigma_n^2)} \\ &\approx \frac{\mu_n^2(1-d)^2}{0.5\sigma_n^2(1+d^2)} \end{aligned} \tag{A4}$$

A1.b Specific LTD: response to noisy stored patterns

In this case, the noisy bits, constituting a fraction α of a stored pattern, produce a (fractional) response indistinguishable from that to a novel pattern, whereas the other non-noisy fraction *(1-α)* produces a response depressed by the factor *d* as above, yielding a mean response of

$$\mu_s \approx \alpha\mu_n + (1-\alpha)d\mu_n = (\alpha + (1-\alpha)d)\mu_n\,. \quad \text{(A5)}$$

Because the noisy bits are random and uncorrelated, we also obtain

$$\sigma_s \approx (\alpha + (1-\alpha)d)\sigma_n\,. \quad \text{(A6)}$$

Substituting Eqs. (A5) and (A6) in (A1) yields for the s/n ration of the response to noisy stored patterns

$$\begin{aligned} s/n &= \frac{(\mu_s - \mu_n)^2}{0.5(\sigma_s^2 + \sigma_n^2)} \\ &\approx \frac{((1-\alpha)d\mu_n + \alpha\mu_n - \mu_n)^2}{0.5((1-\alpha)d + \alpha)^2\sigma_n^2 + \sigma_n^2)} \\ &\approx \frac{\mu_n^2(1-d)^2(1-\alpha)^2}{0.5\sigma_n^2(1 + ((1-\alpha)d + \alpha)^2)} \end{aligned} \quad \text{(A7)}$$

A1.c Non-specific LTD: responses to stored and noisy stored patterns

In this case, the fraction of noisy bits produces a fractional response that is depressed by a factor d_{leak} with respect to (the fractional response to) novel patterns, yielding

$$\mu_s \approx (1-\alpha)d\mu_n + \alpha d_{leak}\mu_n = (\alpha d_{leak} + (1-\alpha)d)\mu_n\,, \quad \text{(A8)}$$

and, likewise,

$$\sigma_s \approx (\alpha d_{leak} + (1-\alpha)d)\sigma_n\,. \quad \text{(A9)}$$

Substituting Eqs. (A8) and (A9) in (A1) yields for the response to noisy patterns in an ANN trained with nsLTD (and only nearest-neighbor relationship)

$$
\begin{aligned}
s/n &= \frac{(\mu_s-\mu_n)^2}{0.5(\sigma_s^2+\sigma_n^2)} \\
&\approx \frac{((1-\alpha)d\mu_n+\alpha d_{leak}\mu_n-\mu_n)^2}{0.5((1-\alpha)d+\alpha d_{leak})^2\sigma_n^2+\sigma_n^2)} \\
&\approx \frac{\mu_n^2((1-\alpha)d+\alpha d_{leak}-1)^2}{0.5\sigma_n^2(1+((1-\alpha)d+\alpha d_{leak})^2)}
\end{aligned}
\tag{A10}
$$

Similar formulas can be derived for noise and nsLTD extending to a span of two or three neighbors on either side.

A2. Calculation of the ANN response to novel patterns using specific LTD

The above expressions (A7) and (A10) still contain the factor μ_n^2/σ_n^2, which we here calculate explicitly. Let us first recall that μ_n and σ_n are the mean and standard deviation of the ANN response to patterns containing a number of fN ON bits (density f times input number N), and hence that they must scale as fN and $\sqrt{fN}$, respectively, to the mean (μ_w) and standard deviation (σ_w) of the *weight* distribution, hence

$$\frac{\mu_n^2}{\sigma_n^2} = fN\frac{\mu_w^2}{\sigma_w^2}. \tag{A11}$$

We can now calculate μ_w and σ_w by assuming that the number of times a synapse is hit by an ON bit (and hence the number of times it is being depressed by d) follows a Poisson distribution. This is a small simplification of what really happens, because we ignore that synapses are activated in spatial patterns; instead we assume that they are hit completely independently with probability

$$\lambda = fNp/N = fp, \tag{A12}$$

and that the probability of being hit n times equals

$$P(n) = e^{-\lambda} \frac{\lambda^n}{n!}. \quad \text{(A13)}$$

(The only purely theoretical implication of this simplification is that number of times a synapse is hit can be greater than the number of stored patterns, but number of times this occurs will be vanishingly small.)

Because each time a synapse is hit, its weight is reduced by the factor *d*, the mean weight is

$$\begin{aligned}
\mu_w &= \sum_{n=0}^{\infty} d^n P(n) \\
&= \sum_{n=0}^{\infty} d^n e^{-\lambda} \frac{\lambda^n}{n!} \\
&= e^{-\lambda} \sum_{n=0}^{\infty} \frac{(d\lambda)^n}{n!} \\
&= e^{-\lambda} e^{d\lambda} \\
&= e^{-\lambda(1-d)}
\end{aligned} \quad \text{(A14)}$$

where we have used the fact that the summation in the third line represents the power series expansion of $e^{d\lambda}$.

The variance of the weight distribution is

$$\sigma_w^2 = < w^2 > - \mu_w^2, \quad \text{(A15)}$$

where the mean squared weight can be calculated as

$$
\begin{aligned}
<w^2> &= \sum_{n=0}^{\infty}(d^n)^2 P(n) \\
&= \sum_{n=0}^{\infty}(d^n)^2 e^{-\lambda}\frac{\lambda^n}{n!} \\
&= e^{-\lambda}\sum_{n=0}^{\infty}\frac{(d^2\lambda)^n}{n!} \\
&= e^{-\lambda}e^{d^2\lambda} \\
&= e^{-\lambda(1-d^2)}
\end{aligned}
\qquad \text{(A16)}
$$

from which

$$
\begin{aligned}
\sigma_w^2 &= <w^2> - \mu_w^2 \\
&= e^{-\lambda(1-d^2)} - e^{-2\lambda(1-d)}
\end{aligned}
\qquad \text{(A17)}
$$

Taken together

$$
\begin{aligned}
\frac{\mu_w^2}{\sigma_w^2} &= \frac{e^{-2\lambda(1-d)}}{e^{-\lambda(1-d^2)} - e^{-2\lambda(1-d)}} \\
&= \frac{1}{e^{\lambda(1-d)^2} - 1}
\end{aligned}
\qquad \text{(A18)}
$$

and, using (A12),

$$
\frac{\mu_n^2}{\sigma_n^2} = fN\frac{1}{e^{\lambda(1-d)^2} - 1} \qquad \text{(A19)}
$$

so that, combining Eqs. (A4), (A12) and (A19), the s/n for (non-noisy) stored patterns is

$$
s/n = \frac{\mu_n^2}{0.5\sigma_n^2}\frac{(1-d)^2}{(1+d^2)} = \frac{2fN}{(e^{fp(1-d)^2}-1)}\frac{(1-d)^2}{(1+d^2)} \qquad \text{(A20)}
$$

A3. Calculation of the ANN response to novel patterns using nonspecific LTD (nsLTD)

To calculate the mean μ_w and the standard deviation σ_w of the weight distribution that is obtained after the patterns have been stored with nsLTD, we assume that two independent Poisson processes are at work: the first one represents the depression of the activated synapses by a factor of d and with probability $\lambda_1 = fNp / N = fp$ as before (section A2), the second one represents the depression of the neighboring synapses by a factor of d_{leak} and with probability λ_2 (the latter being $2\lambda_1$ for a purely nearest neighbor relationship). The two processes are spatially correlated within a single pattern, but after a hundred random patterns have been stored in the ANN this spatial relationship is blended to such a degree that the number of times a synapse has been hit by the first process (depressing central synapses) can be regarded as being independent of the hitting of the same synapse by the second process (depressing neighboring synapses).

Under these assumptions the mean and variance of the synaptic weight w will follow the classical rules for the product of two random variables, in this case the weights w_1 and w_2. Hence,

$$\mu_w = \mu_{w,1}\mu_{w,2} \tag{A21}$$

and

$$\sigma_w^2 = \sigma_{w,1}^2\sigma_{w,2}^2 + \sigma_{w,1}^2\mu_{w,2}^2 + \mu_{w,1}^2\sigma_{w,2}^2 . \tag{A22}$$

In the special case where nsLTD depresses the central synapse to the same degree as LTD, it can easily be shown that the s/n for non-noisy patterns is always smaller with nsLTD than with LTD (see the zero-noise s/n values in Fig. 3 of main text). Indeed, if μ and μ_L (σ and σ_L) denote the mean (standard deviation) of the

weight distributions resulting from the depression of the central and neighboring (leaked) synapses, respectively, then the s/n ratio for nsLTD is proportional to

$$\frac{\mu^2\mu_L^2}{\sigma^2\sigma_L^2+\sigma^2\mu_L^2+\mu^2\sigma_L^2}=\frac{\mu^2}{\sigma^2}\frac{\mu_L^2}{(\sigma_L^2+\mu_L^2+\mu^2\frac{\sigma_L^2}{\sigma^2})} < \frac{\mu^2}{\sigma^2}, \tag{A23}$$

and hence always smaller than the s/n ratio obtained with LTD.

To recapitulate, the mean and variance of the weight distribution, and by extension of the ANN response to random non-stored patterns, can be calculated using the formulae derived in sections A2 (for LTD) and A3 (for nsLTD). From these responses to novel patterns, the responses to stored and noisy stored patterns can be derived through multiplication by the additional factors that have been derived in section A1. Figure 3*B* in the main text illustrates the good matching between the numerically and analytically calculated signal-to-noise ratios.

A4. The ANN response after combined LTD and LTP

Here we consider the case that, as before, the synapses activated by the ON bit of a pattern undergo LTD, and that, in addition, the non-hit synapses undergo a small amount of LTP. The analyses of sections A1 (responses to noisy patterns) and A3 (training with nsLTD) can be transferred almost without modification to the present case, so we limit our analysis here to the calculation of the ANN response to non-noisy patterns after training with standard LTD combined with LTP. We start from Eq. A11, and will number the equations with a suffix 'a' to distinguish them from the corresponding equations of section A2. The major difference will be in the choice of weight distribution: instead of taking a Poisson distribution for the number of times a

synapse has been hit during training (and hence undergone LTD), we now assume that the distribution of times a synapse has undergone LTD and LTP follows a binomial distribution.

As above, Eq. A11, The response to novel patterns can be expressed in terms of the weight distribution as

$$\frac{\mu_n^2}{\sigma_n^2} = fN\frac{\mu_w^2}{\sigma_w^2} \tag{A11a}$$

where N denotes the number of inputs to the ANN, and f the fraction of ON bits in a pattern (density).

Then the probability of a synapse being hit n times (LTD with weight factor d_-) and not-hit p-n times (LTP with weight factor d_+) is (p being the number of stored patterns)

$$P(n, p-n) = \binom{p}{n} f^n (1-f)^{p-n} \tag{A13a}$$

The mean of the distribution can be calculated as:

$$\begin{aligned}
\mu_w &= \sum_{n=0}^{p} P(n, p-n)(d_-)^n (d_+)^{(p-n)} \\
&= \sum_{n=0}^{p} \binom{p}{n} f^n (1-f)^{p-n} (d_-)^n (d_+)^{(p-n)} \\
&= \sum_{n=0}^{p} \binom{p}{n} (fd_-)^n ((1-f)d_+)^{p-n} \\
&= (fd_- + (1-f)d_+)^p
\end{aligned} \tag{A14a}$$

Most natural would be to assume that the average weight does not change during learning, hence

$$fd_- + (1-f)d_+ = 1 \tag{A14b}$$

which trivially gives μ_w=1, because all weights had been initialized at unity.

As before with the Poisson distribution (A15), the variance of the weight distribution can be calculated as

$$\sigma_w^2 = \langle w^2 \rangle - \mu_w^2$$

We have to calculate

$$\begin{aligned} \langle w^2 \rangle &= \sum_{n=0}^{p} P(n, p-n)(d_-)^{2n}(d_+)^{2(p-n)} \\ &= \sum_{n=0}^{p} \binom{p}{n} f^n (1-f)^{p-n} (d_-)^{2n}(d_+)^{2(p-n)} \\ &= (fd_-^2 + (1-f)d_+^2)^p \end{aligned} \tag{A16a}$$

from which

$$\begin{aligned} \sigma_w^2 &= \langle w^2 \rangle - \mu_w^2 \\ &= (fd_-^2 + (1-f)d_+^2)^p - 1 \end{aligned} \tag{A17a}$$

Taken together

$$\frac{\mu_w^2}{\sigma_w^2} = \frac{1}{(fd_-^2 + (1-f)d_+^2)^p - 1} \tag{A18a}$$

and, using (A14b) above,

$$\frac{\mu_n^2}{\sigma_n^2} = fN \frac{1}{(fd_-^2 + (1-f)d_+^2)^p - 1} \tag{A19a}$$

so that, combining (A4), (A12) and (A19a), the s/n for (non-noisy) stored patterns is given by

$$s/n = \frac{\mu_n^2}{0.5\sigma_n^2}\frac{(1-d)^2}{(1+d^2)}$$
$$= 2fN\frac{1}{((fd_-^2+(1-f)d_+^2)^p-1)}\frac{(1-d)^2}{(1+d^2)} \qquad \text{(A20a)}$$

Note that this formula (A20a) for combined LTD and LTP has the same exponential dependency on p (the number of stored patterns) in the denominator as formula (A20) for LTD alone. Hence adding LTP does not qualitatively change such derived quantities as the storage capacity of the ANN.

A5. Assessment of the storage capacity of the ANN using specific LTD

We here compare the storage capacity of the ANN with the capacity of PC perceptron model analyzed by Brunel et al. 2004.

These authors defined a reliability parameter ρ,

$$\rho = \frac{\kappa}{\bar{w}\sqrt{fN(1-f)}} \qquad \text{(A24)}$$

which expresses the distance κ between the threshold (θ) and the center of the input distribution. This distance is measured in units of standard deviation of the (here binomial) input distribution (Fig. A1 *A*). The parameter that best fitted their experimental data (EPSPs) assumed ρ = 2, indicating that the threshold is two standard deviations below the mean (giving 95% correct classifications). In order to compare this metric with the s/n ratio used in the present study, let us assume for

simplicity that the stored and novel patterns generate responses that show the same spread around their mean, $\sigma_s = \sigma_n$, so that

$$\begin{aligned} s/n &= \frac{(\mu_s - \mu_n)^2}{0.5(\sigma_s^2 + \sigma_n^2)} \\ &= \frac{\mu_s - \mu_n}{\sigma_n} \end{aligned} \qquad \text{(A25)}$$

In this case the optimal threshold position is halfway in between the means. Furthermore, to achieve the same reliability, the respective means should be two standard deviations above (below) the threshold (Fig. A1 *A* bottom), yielding an s/n of 4.

Figure A1 *B* plots the value *p* (number of stored patterns) that yields, according to Eq (A20), an s/n of 4. The free parameters are the sparsity *f* (fixed fraction of ON bits in a pattern, horizontal axis), and the degree *d* of synaptic depression during learning (different curve styles as indicated in legend). Note that for realistic pattern densities *f* the present analysis predicts a similar capacity of 40000 patterns as that predicted by Brunel et al. (2004). There are however two important differences between these two studies. First, all stored patterns in our present study activate exactly the same number *fN* of synapses, whereas in Brunel et al. this represented only the average of activated synapses. Secondly, in contrast to Brunel et al. (2004), our capacity metric counts only explicitly stored patterns, discounting the novel patterns that nevertheless are properly classified.

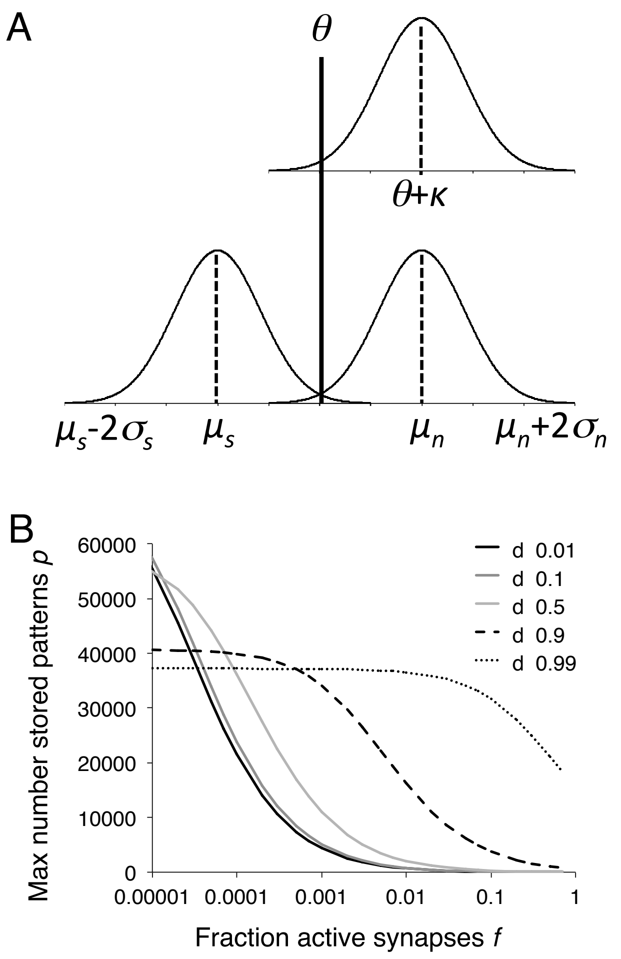

Figure A1 | Assessment of the storage capacity of the ANN for random (non-noisy) patterns. (A) Comparison of the discrimination threshold ϕ used by Brunel et al. 2004 (κ is at the center of the binomial input distribution) (*upper panel*), and the threshold used to calculate the present storage capacity in panel B (ϕ now separates the ANN response distributions to novel (subscript n) and stored patterns (subscript s)) (*lower panel*). An s/n ratio of 4 corresponds to 95 % correct classifications as in Brunel et al. (2004). (B) Storage capacity derived from evaluating Eq. A20, given as the number p of stored patterns that yields an s/n of 4 for varying densities of ON bits (f on horizontal axis) and strengths of LTD (depression factor d in legend).

Supplementary References

Brunel N, Hakim V, Isope P, Nadal JP, Barbour B (2004) Optimal information storage and the distribution of synaptic weights: perceptron versus Purkinje cell. Neuron 43:745-757.

de Sousa G, Maex R, Adams R, Davey N, Steuber V (2014) Synaptic plasticity and pattern recognition in cerebellar Purkinje cells. In: The Computing Dendrite (Cuntz, H. et al., eds), pp 433-448: Springer.

de Sousa G, Maex R, Adams R, Davey N, Steuber V (2015) Dendritic morphology predicts pattern recognition performance in multi-compartmental model neurons with and without active conductances. J Comput Neurosci 38:221-234.

Gutierrez-Castellanos N, Da Silva-Matos CM, Zhou K, Canto CB, Renner MC, Koene LM, Ozyildirim O, Sprengel R, Kessels HW, De Zeeuw CI (2017) Motor learning requires Purkinje cell synaptic potentiation through activation of AMPA-receptor subunit GluA3. Neuron 93:409-424.

Luthman J, Adams R, Davey N, Maex R, Steuber V (2009) Decoding of Purkinje cell pauses by deep cerebellar nucleus neurons. In: BMC Neurosci, vol. 10 (Supp 1).

Marr D (1969) A theory of cerebellar cortex. J Physiol 202:437-470.

Person AL, Raman IM (2011) Purkinje neuron synchrony elicits time-locked spiking in the cerebellar nuclei. Nature 481:502-505.

Schonewille M, Belmeguenai A, Koekkoek SK, Houtman SH, Boele HJ, van Beugen BJ, Gao Z, Badura A, Ohtsuki G, Amerika WE, Hosy E, Hoebeek FE, Elgersma Y, Hansel C, De Zeeuw CI (2010) Purkinje cell-specific knockout of the protein phosphatase PP2B impairs potentiation and cerebellar motor learning. Neuron 67:618-628.

Steuber V, Mittmann W, Hoebeek FE, Silver RA, De Zeeuw CI, Hausser M, De Schutter E (2007) Cerebellar LTD and pattern recognition by Purkinje cells. Neuron 54:121-136.

van Beugen BJ, Gao Z, Boele HJ, Hoebeek F, De Zeeuw CI (2013) High frequency burst firing of granule cells ensures transmission at the parallel fiber to

purkinje cell synapse at the cost of temporal coding. Front Neural Circuits 7:95.

Walter JT, Khodakhah K (2009) The advantages of linear information processing for cerebellar computation. Proc Natl Acad Sci U S A 106:4471-4476.

Wilms CD, Häusser M (2015) Reading out a spatiotemporal population code by imaging neighbouring parallel fibre axons in vivo. In: Nat Commun, vol. 6, p 6464.
